# Molecular epidemiology of *Plasmodium vivax* in Latin America: polymorphism and evolutionary relationships of the circumsporozoite gene

**DOI:** 10.1186/1475-2875-12-243

**Published:** 2013-07-15

**Authors:** Lilia González-Cerón, Jesus Martinez-Barnetche, Ciro Montero-Solís, Frida Santillán, Aida M Soto, Mario H Rodríguez, Benjamin J Espinosa, Octavio A Chávez

**Affiliations:** 1 Centro Regional de Investigación en Salud Pública, Instituto Nacional de Salud Pública, Tapachula, Chiapas, Mexico; 2 Centro de Investigación en Enfermedades Infecciosas, Instituto Nacional de Salud Pública, Cuernavaca, Morelos, Mexico; 3 Pan American Health Organization Office, Managua, Nicaragua; 4 Naval Medical Research Unit - 6, Lima, Peru; 5 Vector Control Program, Silais Chinandega, Ministry of Health, Chinandega, Nicaragua

**Keywords:** *Plasmodium vivax*, Circumsporozoite protein, Gene polymorphism, Mismatch distribution, Phylogeny, Repeat allelic types, Mexico, Nicaragua, Peru

## Abstract

**Background:**

The origins and dispersal of *Plasmodium vivax* to its current worldwide distribution remains controversial. Although progress on *P. vivax* genetics and genomics has been achieved worldwide, information concerning New World parasites remains fragmented and largely incomplete. More information on the genetic diversity in Latin America (LA) is needed to better explain current patterns of parasite dispersion and evolution.

**Methods:**

*Plasmodium vivax* circumsporozoite protein gene polymorphism was investigated using polymerase chain reaction amplification and restriction fragment length polymorphism (PCR-RFLP), and Sanger sequencing in isolates from the Pacific Ocean coast of Mexico, Nicaragua, and Peru. In conjunction with worldwide sequences retrieved from the Genbank, mismatch distribution analysis of central repeat region (CRR), frequency estimation of unique repeat types and phylogenetic analysis of the 3′ terminal region, were performed to obtain an integrative view of the genetic relationships between regional and worldwide isolates.

**Results:**

Four RFLP subtypes, vk210*a*, *b*, *c* and *d* were identified in Southern Mexico and three subtypes vk210*a*, *e* and *f* in Nicaragua. The nucleotide sequences showed that Mexican vk210*a* and all Nicaraguan isolates were similar to other American parasites. In contrast, vk210*b*, *c* and *d* were less frequent, had a domain ANKKAEDA in their carboxyl end and clustered with Asian isolates. All vk247 isolates from Mexico and Peru had identical RFLP pattern. Their nucleotide sequences showed two copies of **GGQAA**GGNAANKKAGDAG**A** at the carboxyl end. Differences in mismatch distribution parameters of the CRR separate vk247 from most vk210 isolates. While vk247 isolates display a homogeneous pattern with no geographical clustering, vk210 isolates display a heterogeneous geographically clustered pattern which clearly separates LA from non-American isolates, except vk210*b, c* and *d* from Southern Mexico.

**Conclusions:**

The presence of vk210*a* in Mexico and vk210*e*, *f* and *g* in Nicaragua are consistent with other previously reported LA isolates and reflect their circulation throughout the continent. The vk210*b, c* and *d* are novel genotypes in LA. Their genetic relationships and low variability within these vk210 and/or within the vk247 parasites in Southern Mexico suggest its recent introduction and/or recent expansion to this region. The global analysis of *P. vivax csp* suggests this parasite introduction to the region and likely LA by different independent events.

## Background

*Plasmodium vivax,* is the most widely distributed malaria parasite in Meso- and South America [1], producing 80–90 million cases per year in these regions [2]. Although in Mesoamerica, malaria transmission has significantly decreased during the last decade [3,4], it persists with fluctuations in several regions. In Mexico, the number of cases peaked during 1998–1999, followed by a gradual decrease until in 2005, when hurricane Stan devastated the southern region resulted in a significant increase in cases in 2006, 2007 and 2008 [5]. Malaria cases in Nicaragua followed a similar gradual pattern, from 23,878 cases in 2000 to 762 in 2008. In Peru, the region of Piura experiences a comparatively high proportion of malaria cases in the coast and a *P. vivax* outbreak occurred in Sullana province during 2008, while surrounding zones experienced lower rates of transmission [3].

The distribution of *P. vivax* populations is controversial; some studies indicate a geographical compartmentalization of the New from Old World parasites [6], but this is not supported by others [7]. Consistently, population genetics of parasites in temperate zones from China suggest an ancient population expansion [8]. Although recent relevant progress on genetic features of the parasite has been achieved in various geographical regions worldwide [8-11], information from endemic areas of the Americas remains largely incomplete. Epidemiological and genetic studies covering malaria-endemic countries in the region could provide a better understanding of parasite transmission and dispersion [9].

*Plasmodium* sporozoites are covered by the circumsporozoite protein (CSP). This protein has multifunctional roles, including sporozoite maturation and salivary gland invasion in mosquitoes and hepatocyte invasion in humans [12]. Motivated by its longstanding as a vaccine candidate, the CSP gene polymorphism has been investigated in parasites from different geographic regions and used to elucidate evolutionary dynamics [13-15]. All CSPs present a central repeat region (CRR) and two conserved domains RI and RII located in the amino and carboxyl ends, respectively. Two main *P. vivax* CSP CRR phenotypes have been described: vk210 [GDRA(A/D)GQPA] [16] and vk247 [ANGA(G/D)(N/D)QPG] [17]. In southern Mexico, Pvs25/28 ookinete surface protein alleles are partially linked to vk210 and vk247 CSP phenotypes, and associated to the parasite infectivity to the local mosquito vectors *Anopheles pseudopuntipennis* and *Anopheles albimanus*[18,19].

The two *P. vivax* CSP main CRR genotypes have been reported at variable frequencies in most malaria-endemic regions, including southern Mexico and Peru [20-28]. The vk210 genotype predominates in many affected areas of Brazil and vk247 has been limited to some regions in mixed *csp* genotype infections [14,29,30]. Recently, the predominance of vk247 parasites and a high level of genetic polymorphism of the *csp* gene were documented on the Pacific Ocean coast of Western Colombia [31]. In the present study, different molecular and bio-informatics approaches were used to analyse *P. vivax csp* diversity and gene polymorphism of isolates from the Pacific Ocean coast of Mexico, Nicaragua and Peru. Their comparison with other isolates from Latin America (LA) and the rest of the world (OA) identified common genetic features in a group of vk210 LA isolates and the recent introduction and/or dispersal of novel vk210 and vk247 genotypes mainly in the Southern Mexican region.

## Methods

### Blood sample collection and geographic origin

**Figure 1:**
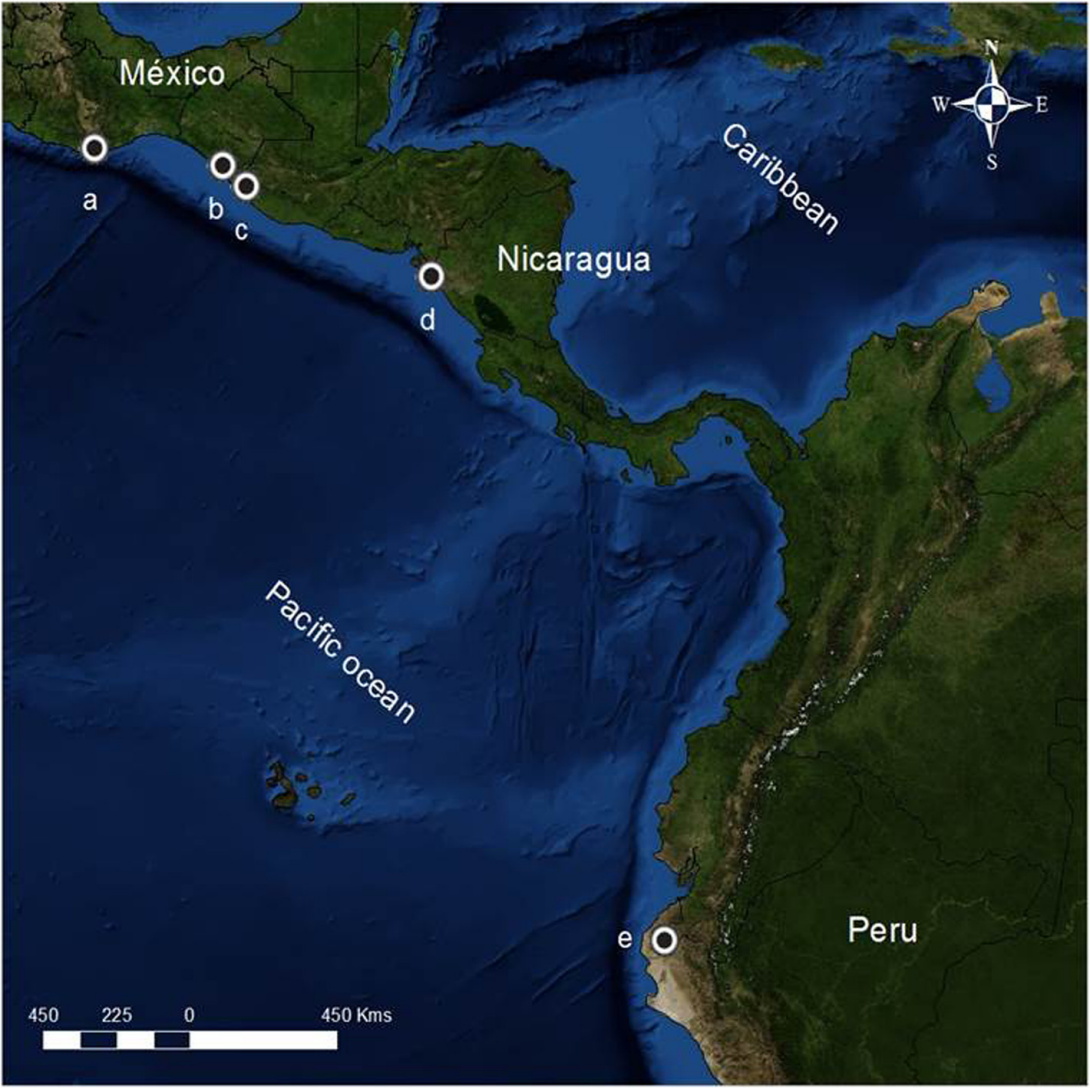
**Study sites. (a)** Pochutla municipality, Oaxaca, Mexico; **(b)** Mapastepec; **(c)** Tapachula municipality and surrounding villages, Chiapas, Mexico; **(d)** Chinandega department, Nicaragua; **(e)** Sullana, Piura, Peru.

Following informed consent, infected blood samples were obtained from symptomatic patients living in Tapachula municipality and neighbouring villages in Southern Chiapas, Mexico. Patients were diagnosed by microscopy, using Giemsa-stained thick and thin blood smears. Two groups of samples were collected; one batch of 70 infected whole-blood samples during 2002–2005 and a second batch of 400 infected capillary blood samples dried on filter paper (Whatman No. 2) were collected between 2006 and 2008. Another three samples were collected in other locations in Southern Chiapas [32,33]. Two other samples were obtained from San Pedro Pochutla, Oaxaca, south-west Mexico in 2007. Other infected dried bloods were collected from affected areas of Nicaragua and Peru. Thirty-seven blood samples were collected in Chinandega, Department of Nicaragua (five from Chinandega town (departmental seat), nine samples from Chichigalpa, 15 from El Viejo, two from Posoltega and six from El Realejo municipalities) from August 2006 to December 2007. The communities are located within 25 km of each other. Nine infected blood samples were obtained from Sullana, Piura, Peru in May, 2008 (Figure 1).

### Polymerase chain reaction and restriction fragment length polymorphism (PCR-RFLP) analysis

PCR-RFLP was used to examine the *csp* polymorphism of all *P. vivax* samples. DNA from blood samples was extracted using the QIAamp® DNA mini kit, as per the manufacturer instructions (Qiagen, CA, USA). Briefly, the *csp* gene amplification was standardized with primers CSPf1 (5′-cgc act gcg ggc aca atg tag atc-3′) and CSPr1 (3′-ggt tac act gca tgg agt cc-5′). The PCR reaction mix was prepared as follows: GoTaqFlexi 1X Buffer, 2 mM magnesium chloride, 0.2 mM dNTPs (Invitrogen, 10297018 Carlsbad, CA, USA), 10 μM each oligonucleotide, 5 U GoTaq® Flexi DNA polymerase (Promega, Madison WI, USA) and ~100 ng of genomic DNA in a 20 μl final volume. PCR conditions were 95°C for 3 min, followed by 35 cycles of 95°C for 30 sec, 58°C for 30 sec, and 72°C for 2 min, and a final extension at 72°C for 10 min. Samples collected as blood spots on filter paper were processed using an alternative nested PCR with primers CSP-f2 (Region I) (5′-aat aag ctg aaa caa cca-3′) and Pv9a (next to region II) (5′-gcc aac ggt agc tct aac ttt- 3′) [24] for the second round amplification. An aliquot (2 μl) of the first amplification sample was added to the second PCR reaction. PCR conditions for the second amplification were the following: first cycle at 95°C for 3 min, followed by 35 cycles each at 95°C for 30 sec, then 57°C (for first PCR) for 30 sec and 72°C for 1 min, followed by a final extension at 72°C for 10 min, using the thermocycler MyCycler (Biorad, Hercules, USA).

The PCR products were analysed using differential restriction enzyme digestion [34]. Enzymes Alu I (New England Biolabs, Beverly, MA, USA) and BstI (Promega, Madison, WI, USA) cut only the *csp* repeat Vk247 and Vk210, respectively. DNA fragments were resolved in a 1.5% agarose gel, visualized in a UV-transilluminator (LMS-20E a 254 NM and photographed with a Digital photo-documentation system BioDoc-it™ (UVPInc, Upland, California, USA).

### Cloning and sequencing

*Plasmodium vivax* isolates producing different vk210 and vk247 PCR molecular size amplified products and RFLP patterns from different geographic origins were selected for cloning and sequencing to analyse their nucleotide and amino acid sequences. The first PCR amplification yielded sufficient product to carry out the cloning using the TOPO TA cloning® kit with pCR®2.1-TOPO® according to manufacturer instructions (Invitrogen, Carlsbad, CA, USA). Some of the samples collected on filter paper were not amplified using this methodology (*csp* genes from isolates Mxch10, Mxch11, Mxch13 and Mxch14, MxO and those from Nicaragua). A modified nested PCR amplification was carried out before the ligation reaction using the following modification: 95°C for 3 min, 10 cycles of 95°C for 30 sec, 50°C for 30 sec and 72°C for 2 min followed by 25 cycles of 95°C for 30 sec, 57°C for 30 sec and 72°C for 2 min and, followed by a final extension of 72°C for 10 min. Modification of blunt-ended PCR products was carried out using the Qiagen A-Addition kit as per manufacturer instructions (Qiagen GmbH, Hilden, Germany).

At least two or three different colonies containing the cloned product of each *csp* allele were sequenced from both directions, using the primers described above. The sequences were obtained using a sequencer ABI PRISM® 3100 Genetic Analyzer at the National Institute for Public Health and the Perkin Elmer/Applied Biosystems 3730 of Biotechnology Institute-UNAM in Cuernavaca, Morelos, Mexico. Some short amplified fragments had few repeat units and were discarded as they could be the result of polymerase failure to amplify the whole repeat fragment.

### Data analysis

The RFLP patterns were ordered by the vk genotype accompanied by a lowercase letter (*a, b, c, d*, etc.). The quality of the electropherogram of each DNA sequence was analysed with both Phred-Phrap-Consed [35] and by manual inspection. The resulting nucleotide sequences were aligned using the six-frame and CLUSTALW (v.3.2) programs, available at the San Diego Supercomputer Center worldwide servers [36]. The allelic consensus sequences were prepared using upstream and downstream nucleotide sequences, both overlapping beyond the central repeat fragment (from RI to RII domains and the other way round). Their nucleotide and amino acid polymorphism were compared to sequences from isolates within and among their geographic origin and to other *P. vivax csp* nucleotide sequences available in databases. Comparison was carried out using the BLASTN and BLASTX v.2.2.17 program [37] available at the NCBI [38]. The complete gene sequences obtained were submitted to the NCBI [Gen Bank accessions: JQ511263.1-JQ511286.1].

The evolutionary and genetic CSP relationship was investigated by carrying out an analysis of the mismatch distribution of the CRR and a phylogenetic relationship of the 3′ terminal nucleotide sequence. The sequences obtained in the present study along with other vk210 and vk247 alleles from America and other geographic origin obtained from the Gen Bank TM (NCBI) were analysed. The *csp*-vk210 group sequences were from El Salvador (XM_001613018.1), Honduras (DQ156131.1), Colombia (GU339072.1, GU339085.1), Brazil (M11926.1, DQ156132.1, DQ978648.1, DQ978651.1, DQ978656.1- DQ978658.1, DQ978675.1, EU401924.1, FJ845386.1, FJ845388.1, FJ845389.1, FJ845390.1), Iran (AY367278.1, AY367286.1, AY443706.1, AY443720.1, AY632256.1, AY632287.1, AY632320.1, AY632325.1), India (EU401926.1, FJ491100.1, FJ491117.1, FJ491119.1, FJ491128.1, DQ156140.1), Thailand (M28746.1), South Korea (DQ859754.1, DQ859760.1, DQ859768.1), North Korea (M20670.1, AF316580.1, AF316581.1), Korea (DQ156137.1), Indonesia (DQ156135.1, EU401927.1), Philippines (U08980.1), Solomon (U08982.1), New Guinea (EU401925), Gabon (U09737), Vietnam (EU401929) and Mauritania (AY674050.1).

The *csp*-vk247 group sequences were from Thailand (M28745.1), Brazil (M69062.1), North Korea (EU401928.1), Colombia (GU339063.1- GU339065.1, GU339067.1- GU339071.1, GU339075.1, GU339076.1, GU339078.1, GU339079.1, GU339082.1, GU339084.1, JN689932.1, JN689933.1), Iran (AY632316.1, AY632330.1, AY632294.1, AY632298.2, AY632299.2, AY443710.2, AY632330.1), Bangladesh (AY843440.1) and Vietnam (DQ156141.1; EU401930.1).

As vk- references, *csp* sequence of *Plasmodium simium* I and II isolated from Brazil were incorporated in the analysis: type vk247 (L05069.1) and type vk210 (L05068.1), respectively.

### Mismatch distribution analysis of the CRR sequence

A mismatch distribution analysis [13] was carried out in isolates sequenced here, compared to others from LA and OA regions. For an automated identification of vk210 or vk247 repeat allotypes [39] within each *csp* nucleotide sequence, program HMMER3 were used [40]. First, a HMM profile was created with *hmmbuild* using as input the aligned unique repeat nucleotide (*rat*) sequences described by Patil *et al.*[14] or from vk247 *rat* sequences identified by eye. The hmm profile was then validated by searching *rat*s in Patil *et al.*[14] dataset and the Mexican and Peruvian VK247 alignments. A search for *rats* in the GenBank sequences was performed with *hmmsearch* using a per domain inclusion threshold (−−incdomE) of 1.0E-05. The “-A” option was selected as output to obtain the target *rat* sequence as multiple alignment in Stockholm format, which was transformed to FASTA file. Individual *rat* sequences were analysed by eye to confirm that were *bona fide* CRR units [14,16,17,31,41]. For pairwise comparisons between *rats,* all sequence comparisons within a CRR sequence were performed with USEARCH version 5.0 [42] with the “--nousort” option. To obtain the mismatch count for the whole CRR sequence, the global alignment option (“--global”) was enabled. Mismatch counts for each pairwise comparisons within each isolate were retrieved from tabular output specified by the “--blast6out” option. The mean proportion of nucleotide difference for all pairwise comparisons among CRR units (*p*); the proportion of all pairwise comparisons in which *p* was zero (*prop. 0*); the proportion of all pairwise comparisons in which *p* was greater than 0.25 (*prop. >0.25*) the skewness (Pearson asymmetry coefficient) and the variance of the distribution of *p* for all pairwise comparisons were calculated [13,14]. Additionally, the variance of the *p* distribution values for all pairwise comparisons (*σ* (*p*)) was also calculated. Different combinations of MD parameters were plotted to search for clusters. Also, the five mismatch distribution parameters were normalized to its respective highest value and subjected to hierarchical clustering (complete linkage) using uncentred absolute correlation as similarity metric with Cluster 3.0 [43] and visualized in Java TreeView 1.1.6.r2.

The frequency of *P. vivax* unique repeat types at amino acid (RAT) and nucleotide level (*rat*) verified by local BLASTn searches were calculated for each group vk210 and vk247 and by geographic region: LA *versus* OA origins. Two-sample *t*-test for continuous variables and the Pearson chi-squared test for proportions were used to establish significant differences between the groups vk210 *vs* vk247 and within vk210 subgroups: C1 *vs* C2 and C1A *vs* C1B, and for *CSP* RATs and nucleotide sequence usage (*rats*) between LA *vs* OA isolates, respectively (α = 0.05).

### Phylogenetic analysis

To search similarities at the 3′ terminal variable region of the *csp* gene, an alignment of the nucleotide sequences of the isolates indicated in the previous section, comprising the sequence between last 3′ CRR unit and domain RII, was used to construct a phylogenetic tree using the neighbour-joining method with 1,000 bootstrap replications [44] and conducted in MEGA4 [45].

### Ethical clearance

This study was approved by the Ethical Committee of the Mexican National Institute of Public Health. Samples from Nicaragua and Peru were collected as part of projects approved by the Nicaraguan Ministry of Health and PAHO and the Institutional Review Board of NAMRU-6 in Peru, respectively.

## Results

### DNA amplification and PCR-RFLP analysis of *Plasmodium vivax csp* gene

A total of 521 samples were analysed. Seven different amplified fragment sizes and/or RFLP patterns were identified in 267 vk210 genes, while only one amplified fragment size and RFLP pattern were identified in 288 vk247 genes.

### Samples from Southern Mexico (Chiapas and Oaxaca)

**Figure 2:**
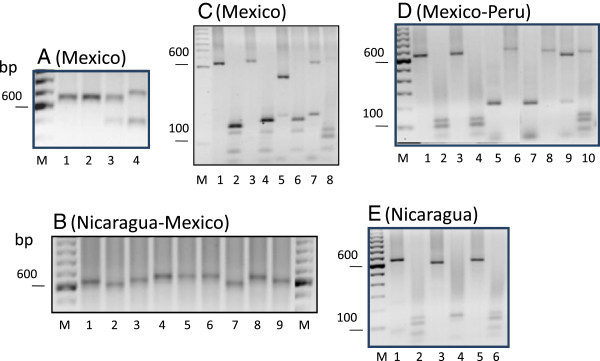
**Molecular size variation and RFLP patterns of the *csp *gene of *Plasmodium vivax *isolates from the central Pacific Ocean coast region of the Americas A and B, PCR amplified product size. A)** Mexican samples; ~700 pb -vk210*a* (lane 1) and ~700 pb -vk210*b* (lane 2) had the same molecular size. Lane 3 and 4, show mixed genotype infections: (vk210*b* and ~500 pb- vk210*c*) and (>700 pb-vk247 and vk210*c*), respectively. **B)** Nicaraguan and Mexican samples; fragments of ~700 bp (lanes 4, 5 and 8; Nir1), ~650 bp (lanes 1, 3 and 9; Nichn), ~600 bp (lanes 2 and 7) and vk210a from Mexico (lane 6) are shown.RFLP patterns: **C)** Mexican samples; vk210*d* (lane 1–2), vk210*b (*Lane 3–4), vk210c +vk247 (lane 5–6), and vk247+vk210*a* (lane 7–8). **D)** Mexican and Peruvian samples; vk210*a* (lanes 1–4, two samples), vk247 (lanes 5–8, two samples: Mexican and Peruvian); and mixed infection Vk210*a*-vk247 (lane 9–10). **E)** Nicaraguan samples; vk210*a* like - Nir1 isolate (lane 1 and 2), vk210*e* - Nichn isolate (lane 3–4), vk210*f* - Nir2 isolate (lane 5 and 6) are shown. **C**, **D** and **E**: odd numbers indicate Alu1 digestion and round numbers indicate BstI digestion. 1% agarose gels and ethidium bromide for staining were used. M, marker 1 kb.

From 475 samples, four different vk210 subtypes were named as *a, b, c* and *d* (Tables 1 and 2). Subtype vk210*a* was present in 208 single and 28 mixed genotype infections; it produced an amplified gene fragment of ~700 bp and a RFLP pattern of ~135/130 and 100 bp. The other subtypes were less frequent, subtype vk210*b* was present in seven single and six mixed *csp* genotype infections*,* and these had a smaller molecular weight to vk210*a* parasites and had a RFLP pattern of ~190/130 bp. Subtype Vk210*c* was present in eight mixed genotype infections, had an amplified nucleotide fragment of ~500 bp and digestion products of ~200/130 bp (Figure 2A and 2C). The subtype vk210*d* was detected in only one isolate (in 2006); it presented an amplified fragment of molecular size similar as vk210*a* (~700 bp) but a different RFLP pattern of ~170/130 bp (Figure 2C). All vk247 isolates from 215 single and 40 mixed genotype infections from Southern Chiapas and the two samples from Oaxaca produced an amplified product of ~710 bp (slightly larger than the vk210*a* fragment size) and the *Bst*I digestion generated products of ~220 bp (Figure 2A and 2D)*.* The *csp* genotype frequency of vk210 and vk247 in single or mixed genotype infections in samples collected during the 2006–2008 period is presented in Table 2. A high percentage of single genotype infections and similar frequency for both vk210 and vk247 genotypes were observed, but a higher frequency of vk210*a* compared to vk247 infections was detected throughout 2008.

**Table 1:** ***Plasmodium vivax *circumsporozoite gene variation in isolates from the central Pacific Ocean coast of Latin America**

**Geographic origin**	**Total (n)**	^**a**^***csp CRR genotype*:**				***Csp*nucleotide sequence:**			
		**Type**	**n**	**PCR Product size (bp)**	**RFLP type**	**Isolate code**	**Date of collection**	^**b, f**^**Gene sequence (bp)**	**# of CRRu**
**Mexico:** Southern Chiapas: (batch 2002–2005)	2 s	vk210	1	~700	*a*	Mxch13	May 5, 2000	657 ^*II*^	20
		vk247	1	^e^>700		Mxch10	March 3, 1999	714	19
	70	vk210	9 s	~700	*a*	Mxch1	Jul 5, 2005	^c^894 ^*I*^	20
			12 m			Mxch2	Oct 3, 2002	“	“
						**Mxch3**	March 10, 2005	“	“
		vk210	7 s	~700	*b*	Mxch4	July 4, 2002	^c^900	18
						**Mxch5**	Feb 4, 2003	“	“
			5 m			**Mxch7**	May 16, 2002	“	“
		vk210	5 m	~500	*c*	**Mxch3**	-	^c^738	12
						**Mxch5**	-	“	“
						**Mxch8**	Nov 17, 2003	“	“
						**Mxch9**	July 4, 2002	“	“
		vk247	33 s	^e^>700	*-*	Mxch6	July 2, 2002	^c^951	19
			20 m			**Mxch7**	-	“	“
						**Mxch8**	-	“	“
						**Mxch9**	-	“	“
						Mxch12	Feb 16, 2004	“	“
						Mxch11	Aug 16, 2004	714	“
^d^(batch 2006–2008)		vk210	(1 s)	~700	*d*	Mxch14	Nov 6, 2006	639	18
Oaxaca	2 s	vk247		^e^>700	*-*	MxO	Feb 22, 2007	714	19
**Nicaragua**	37 s		20	~700	*a*	Nir1	Jan 11, 2007	711 ^*III*^	22
		vk210	12	~650	*e*	Nichn	May 17, 2007	630	19
			5	~600	*f*	Nir2,	Nov 27, 2006	603	18
						Nichg	July 2, 2007	603	“
**Peru**	9 s	vk247		^e^>700	-	Peru1	May, 2008	^c^951	19

s, single *csp* repeat genotype infection ; m, isolates with mixed vk210-vk247 or vk210-vk210 infections (in bold letters).

^a^Nested PCR (fragments amplified with oligonucleotides CSP-f1/CSP-r1 followed by primers CSP-f2/Pv9a).

^b^Only those sequences obtained from the amplified product obtained by the first set of primers (CSP-f1/CSP-r1).

CRRu, central repeat units.

^c^225 nucleotides from the amino end were also obtained for these isolates.

^d^See data of batch 2006–2008 in Table 2.

^e^Molecular size slightly larger and discernible from vk210*a* fragment.

^f^Three different nucleotide sequences ^(*I, II* and *III*)^ were detected for RFLP-vk210*a.*

**Table 2:** **Prevalence of *Plasmodium vivax *circumsporozoite of RFLP genotypes in Southern Chiapas, Mexico during 2006-2008**

	**Year:**							
**RFLP-genotype:**	**2006**		**2007**		**2008**		**Complete period:**	
	**n**	**%**	**n**	**%**	**n**	**%**	**n**	**%**
**Single infections:**	**120**	**95.2**	**131**	**93.4**	**128**	**95.5**	**379**	**95**
Vk247^1^	64	50.8	69	49.2	48	35.8^3^	181	45.25
Vk210*a*	56	44.4	62	44.2	80	59.7^3^	198	49.5
Vk210*d*^2^	1	-	-	-	-	-	1	0.25
**Mixed infections:**	**6**	**4.7**	**9**	**6.4**	**6**	**4.4**	**21**	**5**
Vk247^1^ -210*a*	3		8		5		16	4.0
Vk247^1^ -210*b*	1		-		-		1	0.25
Vk247^1^ -210*c*	1		1		1		3	0.75
**Total**	**126**		**140**		**134**		**400**	

n, number of isolates. %, percentage.

^1^All isolates show similar RFLP pattern as those found for years 2002–2005 in Southern Mexico.

^2^New RFLP pattern was detected in a single isolate.

^3^A significant difference, Z test dependent variables at 95% CI.

### Samples from Nicaragua and Peru

All 37 samples obtained in Nicaragua were single vk210 infections. Three subtypes, *a*, *e* and *f* were identified by the molecular size of the amplified product and RFLP *Alu* I digestion patterns: (Table 1, Figure 2B). Twenty samples (vk210*a*) had similar molecular size and RFLP pattern as previously indicated for Mexican isolates, in 12 (vk210*e*) samples the amplified fragment was of ~650 bp of RFLP pattern ~130 and 105/100 bp and in five samples (vk210*f*) a shorter fragment of ~600 bp was amplified with RFLP pattern of 135/130 and 100 bp. All of them had slightly different RFLP digestion patterns (Figure 2E). These three RFLP patterns were distributed in all sites at Chinandega department. The nine samples from Peru were vk247, and all had a conserved molecular size and RFLP pattern similar to the Mexican vk247 isolates (Table 1, Figure 2D).

### *Csp* nucleotide sequence analysis

From 20 *P. vivax*-infected blood samples, 25 *csp* nucleotide sequences were obtained (Table 1). Sixteen sequences comprised 75 codons at the amino end, the CRR through RII domain. Other nine sequences comprised only from RI to RII domains inclusive.

### Pre repeat 5 ′terminal end polymorphism

Gene sequences were compared between 225 nucleotides of the conserved amino region, including region I of three vk210*a* (isolates Mxch: 1, 2, 3), three vk210*b* (isolates Mxch: 4, 5, 7), four vk210*c* (isolates Mxch: 3, 5, 8, 9) and five Mexican vk247 *csp* (Mxch: 6, 7, 8, 9, 12) and one Peruvian (Peru I). All vk210 and vk247 had identical nucleotide sequence, except for codon 38, which was Asn (AAC) and Gly (GGC) in vk210 and vk247 samples, respectively.

### Polymorphism and mismatch distribution (MD) of the CRR

#### CRR polymorphism

**Figure 3:**
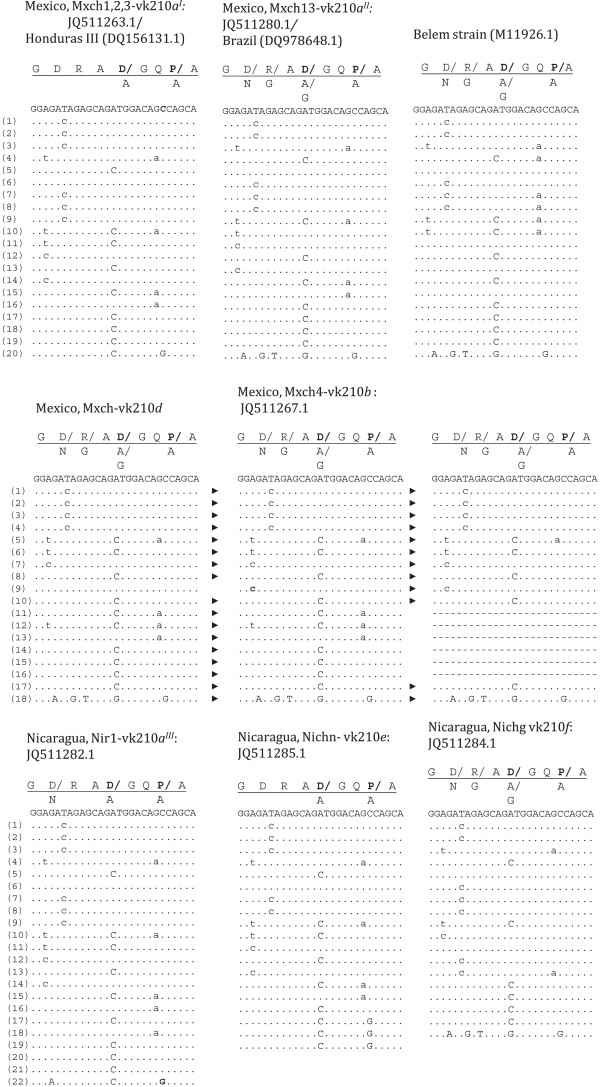
**Comparison of amino acid and nucleotide sequences of the *Plasmodium vivax *circumsporozoite CRR vk210.** The sample code and the Gen Bank accession numbers are indicated at top of each repeat block and those from other isolates worldwide are within parenthesis. The amino acid residues are indicated by capital letters, and those corresponding to the nucleotide sequence are underlined; the amino acid substitutions are indicated underneath. On the left side the repeat units are numbered. Dots indicate absence of nucleotide variation. The non-synonymous nucleotide changes are indicated by upper-case letters. As reference for tandem repeat; Honduras III and Belem strains. The dashed lines indicate missing repeats in the vk210*c* sequences compared to vk210*b* sequence.

Sixteen *csp* vk210 consensus sequences were analysed. The PCR-RFLP subtype vk210*a* produced three different CRR nucleotide sequences named as *a*^*I*^, *a*^*II*^ and *a*^*III*^. *Csp* subtype vk210*a*^*I*^ (Mxch:1, 2, 3) had 20 repeat units; their sequence was identical to that of the Honduras III strain (DQ156131.1), and compared to the Sal I sequence (XM001613018.1) [46] they present a non-synonymous change G→C at the eighth nucleotide codon of the 19th repeat unit (Figure 3). But the nucleotide sequence of isolate vk210*a*^*II*^ (Mxch13) was identical to a previously described Brazilian strain (DQ978648.1) [41]. The *csp* gene of subtypes vk210*b*, *c* and *d* were different to that of Sal I and South American parasites. All one single (Mxch4) and two mixed infections (Mxch5 and Mxch7) vk210*b* sequences examined, had 18 repeats, and were identical among them (Figure 3A). The vk210*c* sequences (isolates Mxch: 3, 5, 8, 9) had 12 repeats and match to the first 10 and the last two repeat units of the 18 repeats of vk210*b* parasites. The vk210*d* (isolate Mxch14) had 18 repeats and differs from vk210*b* by only one nucleotide change at the first codon of the ninth repeat (C→A) (Figure 3).

The *csp* of three different Nicaraguan parasites examined had variable number of repeats and nucleotide substitutions. The *csp* vk210*a*^*III*^ (isolate Nir1), vk210*e* (isolate Nichn) and vk210*f* (isolate Nir2 and Nichg) had 22, 19 and 18, CRR units, respectively (Table 1, Figure 3). The CRR of vk210*a*^*III*^ had a 96% nucleotide identity to the 23 repeats of Brazil I strain (DQ156132.1), and nucleotide variations restricted to the five 3′ terminal repeat units. The *csp* vk210*e* and vk210f were also 97% similar to genes previously described in another Brazilian isolates: O10 CSP (DQ978656.1) that had 19 CRR units and O4 CSP (DQ978651.1) that had 18 CRR units, respectively [41]. In these, nucleotide changes were present in most CRR units.

**Figure 4:**
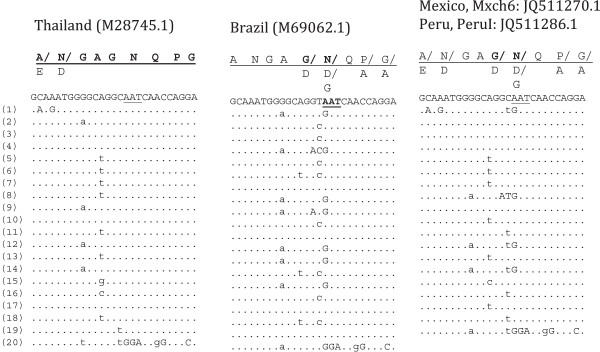
**Comparison of amino acid and nucleotide sequences of the *Plasmodium vivax *circumsporozoite CRR vk247.** The sample code and the Gen Bank accession numbers are indicated at top of each repeat block and those from other isolates worldwide are within parenthesis. The amino acid residues are indicated by capital letters, and those corresponding to the nucleotide sequence are underlined; the amino acid substitutions are indicated underneath. On the left side the repeat units are numbered. Dots indicate absence of nucleotide variation. The non-synonymous nucleotide changes are indicated by upper-case letters. Sequences from isolates Mxch6 and PeruI are shown, which are identical to Iranian (AY632330.1) and Colombian (JN689931.1) isolates and different from sequences of Thailand and Brazilian isolates.

The nine vk247 nucleotide sequences described here had 19 CRR units that were conserved among them. The CRR nucleotide sequences obtained in this study were identical to that of one Colombian (JN689931.1) and one Iranian (AY632330.1) sequences; but the analysis revealed two other different sequences in isolates from Brazil (M69062.1) and Thailand (M28745.1) (Figure 4).

#### MD analysis of the CRR

The MD analysis of worldwide vk210 and vk247 isolates showed low average proportion of mismatches *(p)* (average 0.086 ± 0.01, range 0.05 - 0.15). According to Hughes [13] negative skew values observed for a distinctive group of vk210 LA isolates, including some from Mexico and Nicaragua (Additional files 1 and 2), could be suggestive of ancient duplication events, but are in contradiction with their low *average p*, low *prop. >0.25* and high *prop. 0.* It is reasoned that for very recent duplication events, the σ of *p* distribution (*σ (p)*) would be low. Accordingly, there were a considerable number of LA isolates having negative skew values but low average *p,* indicating very few mutations.

**Figure 5:**
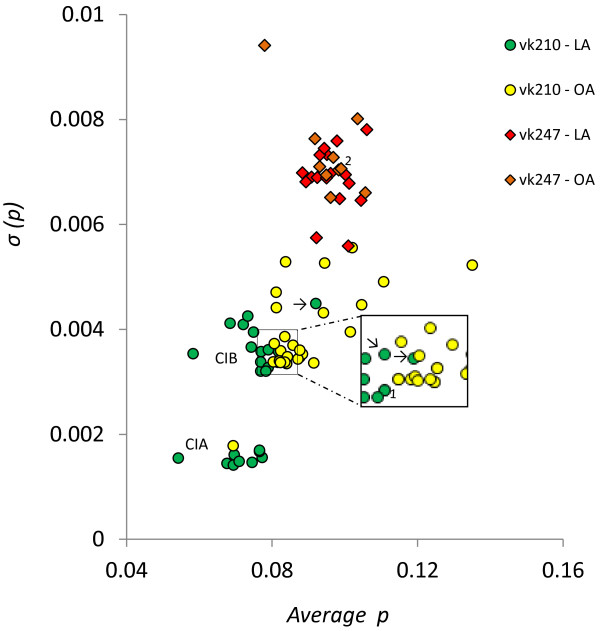
**Mismatch distribution of *Plasmodium vivax *circumsporozoite central repeat sequence: *average p versus σ (p). ****Average p* and its *variance* (σ (*p*)) clearly cluster central sequences and separate vk247 from vk210 sequences. One worldwide group formed by vk247 parasites with the highest *average p* and *σ (p).* Latin American (LA) isolates had a *csp* central repeat of lower *average p* than sequences from outside America (OA; other geographic origins). LA isolates were further separated in two groups by their *p variance*, one with lower σ (*p*) (C1A; Sal I, Honduras III, Mxch3-vk210*a*^*I*^, Nichn, Nir1-vk210*a*^*III*^) than the other (C1B; Mxch13-vk210*a*^*II*^, Nichg). The latter was separated by the *average p* from the OA isolates (C2; Mxch4-vk210*b*, Mxch3-vk210*c*). C2 is composed by a tight group and others of higher *average p* and *σ (p)* were scattered (see also Additional files 1 and 2). Mxch14-vk210*d* and *P. simium* I are marginal between LA and OA isolates. The Mexican vk210*b, c* and *d* are indicated by arrows. ^1^*P. simium* I and ^2^*P. simium* II.

**Figure 6:**
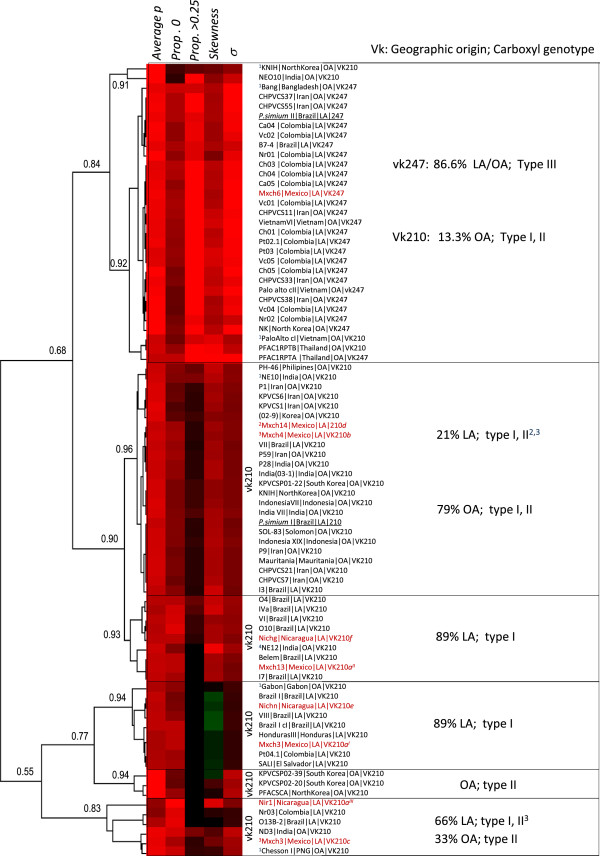
**Hierarchical clustering of the CRR mismatch distribution parameters and its relation to vk genotype, geographic origin and carboxyl end.** The mismatch distribution parameters (*average p, prop. 0, prop. >0.25, skewness and σ (p)* were normalized to the highest value and cluster by uncentred absolute correlation. The frequency of sequences by vk genotype and geographic origin are indicated. Samples from this study are indicated in red. Carboxyl type was indicated by the absence or presence of domain ANKKAEDA as type I and II, respectively, and the presence of ANKKAGDA domain as type III. LA, Latin America. OA, outside Latin America; ^1^ indicate recombinant sequences.

At least three main clusters were identified by comparing different MD parameters, which were more clearly resolved by their *average p* and its σ *(p)* as an alternative measure of MD (Figure 5). Similar clustering was obtained by the hierarchical clustering of normalized MD parameters (Figure 6). Regardless of their geographical origin, all vk247 CRR sequences formed an unique homogeneous cluster (Figures 5 and 6), displaying higher *p*, skew, *prop. >0.25* and *σ (p)* values than most vk210 CRR sequences (Table 3A, Additional file 2). The worldwide vk247 isolates grouped with few vk210 OA isolates, mainly recombinant sequences, except one from Thailand, while vk210 CRR sequences present a heterogeneous clustering with highly correlated sub-clusters: most isolates collected in LA showed lower MD values (except *prop. 0*) (C1) than those from OA (C2) (Figure 5, Table 3B). C1 was further separated in two groups by their *σ (p)* (Figure 5); LA/C1A-vk210 had the lowest *σ (p)* included mainly Mesoamerican isolates (Sal I, Honduras III, Mxch3 (vk210*a*^*I*^), Nichn and Nir1 (vk210*a*^*III*^ and *e* respectively)) but also had four from South America (three from Brazil and one from Colombia) and one from Africa (Gabon isolate). All of the isolates in this group had *prop. >0.25* equal to cero; many had negative skewness, the lowest average *p* and σ *(p)*; LA/sC1B-vk210 includes Mxch13 (vk210*a*^*II*^), Nichg (vk210*f*) and other isolates from South America (Figure 5, Table 3C). However, Mexican isolates vk210*b* with and *c* sequences cluster with OA isolates in C2 (Figure 5) and vk210*d* was marginal between C1 and C2 groups. The hierarchical clustering of normalized MD parameters not only agreed to a great extent to the groups formed by *average p vs* σ *(p)* but also to the carboxyl end genotype (Figure 6). The vk210*b* and *d*, two Brazilian isolates and *Plasmodium simium* I cluster with OA vk210 isolates. The vk210*c* and vk210*a*^*III*^ sequences clustered separately with other OA group and have the highest *prop. 0* values (Figures 5 and 6). In between, two other groups comprised mainly of LA isolates, consistently clustered in C1A and C1B, this sequences of LA parasites do not express the ANKKAEDA domain at carboxyl end, except isolates vk210*b*, *c* and *d* from Southern Mexico as other OA isolates (Figure 6).

**Table 3:** **Statistical differences of mismatch distribution parameters for *Plasmodium vivax csp *CRR by vk genotype and geographic origin**

**Comparison:**		**n**	**Measure**	***average p***	***Prop. 0***	***Prop. >0.25***	***skewness***	***σ (p)***
A:	Worldwide *csp* genotypes:							
	VK210	53	mean	0.082	0.1366	0.0156	0.7693	0.0034
			SD	0.0129	0.0443	0.0194	0.5983	0.0011
	VK247	25	mean	0.0959	0.1371	0.0825	1.2256	0.007
			SD	0.0061	0.0227	0.0092	0.2442	0.0007
	***p***			***0.0001***	***NS***	***0.0001***	***0.0005***	***0.0001***
B:	vk210 worldwide clusters:							
	LA-C1*	21	mean	0.0723	0.1699	0.0062	0.5463	0.0027
			SD	0.0064	0.0434	0.0100	0.7431	0.0011
	OA-C2	30	mean	0.0886	0.1132	0.0223	0.9443	0.0039
			SD	0.0122	0.0300	0.0223	0.4237	0.0008
	***p***			***0.0001***	***0.0001***	***0.0032***	***0.0189***	***0.0001***
C:	vk210 Latin American groups:							
	LA-C1A	10	mean	0.0704	0.1564	0	−0.2242	0.0015
			SD	0.0068	0.0353	0	0.2295	0.0001
	LA-C1B*	11	mean	0.0733	0.1564	0.0117	1.1788	0.0037
			SD	0.0062	0.0334	0.0112	0.1605	0.0003
	***p***			***NS***	***NS***	***0.0038***	***0.0001***	***0.0001***

* The vk210*b*, vk210*c* and vk210*d* showed values more similar to OA than LA isolates and the carboxyl end was closed related to Asian isolates, and to avoid biased, they were not included in this group.

SD, standard deviation.

*P*, significance at 95% (two-sample *t* test).

#### Repeat allotype variation among LA and OA *csp* sequences

**Figure 7:**
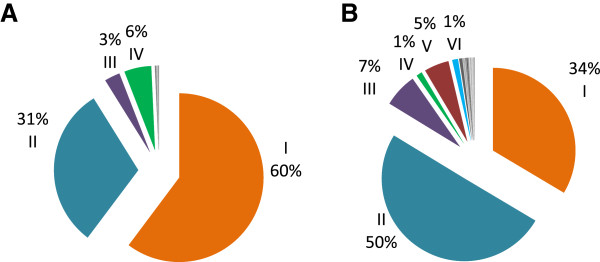
**Repeat allelic type (RAT) frequency of the circumsporozoite central repeats vk210. A)** The 23 Latin American (LA) sequences present seven different RATs; three were exclusive. **B)** The 30 sequences from outside LA (OA) parasites present 17 RATs and 13 were exclusive. RAT I and IV were more frequent in LA than in OA sequences and RAT II and III were more frequent in OA than LA sequences. I. GDRADGQPA; II. GDRAAGQPA; III. GNGAGGQAA; IV. GDRAAGQAA; V. GDGAAGQPA; VI. GNGAGGQPA (see also Additional file 3A).

Nine hundred and fifty repeat units of 27- nucleotides each were identified in 53 vk210 isolates, 23 from LA and 30 from OA origin (Additional file 3). Nucleotide sequences were clustered in 47 unique repeat nucleotide sequence allotype (*rat)* coding for 20 different repeat amino acid allotypes (RAT). Only 14 *rats* were common in parasites of OA and LA origin, and 29 *rats* were exclusive for OA *versus* four *rats* for LA isolates; from Nicaragua and Brazil. RAT I (GDRADGQPA) and IV (GDRAAGQAA) were more frequent in LA isolates than in the OA, while RATs II (GDRAAGQPA), III (GNGAGGQAA) and V (GDGAAGQPA) were more frequent in OA than LA sequences. RATs I and II represent 87% of the total they were highly variable (Figure 7) and coded by 11 and 13 different nucleotide repeat sequences, respectively. Other RATs were coded by three or fewer nucleotide sequences. In LA isolates, alleles I.1, I.3, I.5 were more frequent and alleles I.2, I.3, II.5 and II.6 were less frequent than in isolates from OA. There were some RATs found in only one isolate: three in LA and 11 in OA sequences. A private RAT XIII (GNRAAGQAA) was expressed in a Nicaraguan isolate. RAT IV (GDRAAGQAA) is coded by an unique *rat* in most sequences of group C1A (9 of 11) and three other isolates from Korea. RAT IV differs from RAT II (GDRAAGQPA) by a nucleotide change at the eighth codon GCA→ CCA.

Five hundred and twelve repeat units of 27- nucleotide each were obtained from 26 vk247 isolates; 16 from LA and 10 from OA sequences. Fewer amino acid and nucleotide sequences were present than in vk210 origin (Additional file 4). From 30 *rats* coding for nine RATs only one RAT (I.ANGAGNQPG) was highly variable, coded by 13 different *rats*. All other RATs were coded by four or fewer nucleotide sequences. Consistently with the MD analysis that showed no geographical clustering for vk247 *rats*, there were no significant differences in the presence of any RAT between LA and OA. For RAT I, alleles I.2 was more frequent and I.5 was less frequent in LA isolates than in those from OA origin.

#### Post repeat 3′ terminal end polymorphism and its genetic relationships

**Figure 8:**
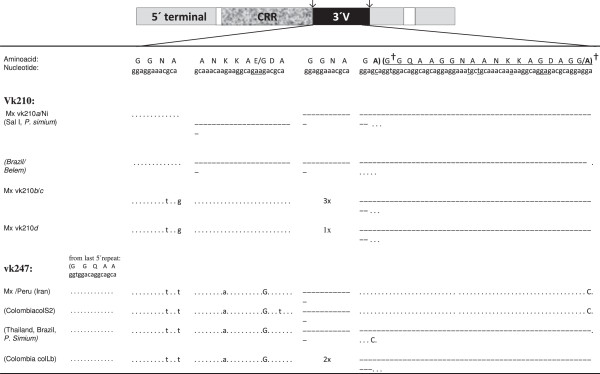
**Comparison of amino acid and nucleotide sequences of the circumsporozoite post repeat 3′ terminal region.** Key differences linked to vk210 or vk247 CR types are shown. The *csp* primary structure indicating the interspecies: conserved RI and RII, white boxes. CRR, central repeat region. The variable 3′ terminal region (3′V) between CR region and the conserved domain GQGQ of the *csp* gene is shown; points indicate absence of nucleotide variation. The dashed lines indicate absence of the fragment. The synonymous and non-synonymous nucleotide changes are indicated by lower and upper case letters respectively. Amino acid substitutions are separated by a slash. Other *csp* sequences of Sal I strain (X_M001613018), Brazil (DQ978648.1), Belem strain (M11926.1), Korean (DQ156137.1) and Colombian isolates ColS2 (JN689932.1); ColLb (JN689933.1) are indicated as reference. In parenthesis two identical fragments of 57 nucleotide, including last codon of Gly or Ala residue†, are indicated. Nd, no determined. Sm, Southern Mexican isolates. Nicaraguan isolates: Nir1, Nir2, Nichn and Nichg.

**Figure 9:**
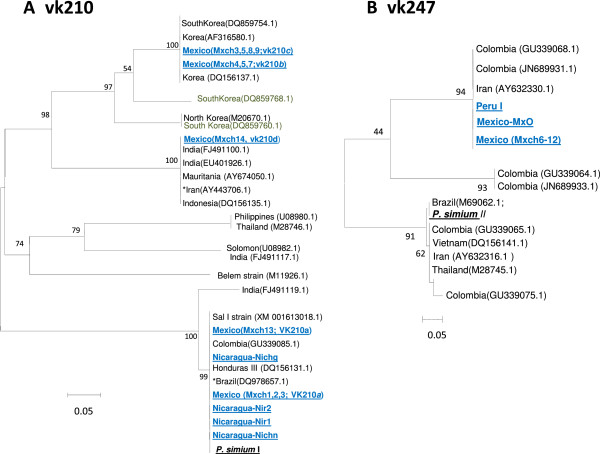
**Phylogenetic relationship of *Plasmodium vivax *circumsporozoite 3′ terminal region.** The vk210*a* from Mexico and those from Nicaragua cluster together with other Latin American (LA) isolates in one branch of the tree. However *csp* sequences vk210 (*b* and *c*) cluster with sequences from Korean parasites. As well, the vk210*d* sequence clustered with parasites from other regions outside LA. All constructions were inferred using the Neighbour-Joining method. The percentages of replicate trees in which the associated taxa clustered together in the bootstrap test (1,000 replicates) are presented next to the branches. Csp sequence analysed in here are in blue.

The nucleotide sequence variation of the 3′ terminal region was sufficient to generate robust phylogenetic trees (Figures 8 and 9). All Nicaraguan (vk210*a*^*III*^, e and *f*) and some Mexican (vk210*a*^*I*^*and a*^*II*^) isolates were similar to the Sal I sequence. The phylogenetic tree confirmed the sequence relationships among Central and South American parasites. These included the Honduras III and Sal I and other from Colombia and Brazil, and all clustered with *P. simium* in one branch of the tree separated from the origin. In contrast, the Mexican vk210*b, c* and *d* types had two synonymous changes in the nucleotide fragment coding for GGNA that flanks the last repeat unit, and it is followed by 57 nucleotides coding the peptide domain ANKKAEDA. The vk210*b* and *c* sequences had a triplet of GGNA flanking the carboxyl end, while vk210*d* had one single copy. These sequences had a Gly residue contiguous to the conserved domain GQGQ. Accordingly, they clustered with vk210 OA isolates having the domain ANKKAEDA on other branches of the phylogenetic tree. The vk210*b* and *c* had identical nucleotide sequences to some Korean isolates and vk210*d* to other OA isolates.

All *csp*vk247 nucleotide sequence from samples collected in Mexico and Peru were conserved and clustered together with Iranian and Colombian *csp* sequences. They present GGNA domains with different synonymous changes than vk210, and display two copies of the domain GGQAA**GGNA**ANKKAGDAGA plus GA residues flanking the GQGQ conserved domain. These sequences were identical to that of an Iranian isolate (AY632330.1) [47] and closely related to recently reported Colombian parasites (JN689931.1, JN689932.1) [31]). All vk210 and vk247 analysed have a conserved fragment of 38 codons between GQGQ and Region II inclusive, and are identical to that of the Sal I strain. The *csp* sequences form OA had more variability in their carboxyl end.

#### Recombinant *csp* nucleotide sequences

No evidence of recombination events [48] were identified in all sequences vk210 or vk247 from Mexico, Nicaragua and Peru, a feature also found in most sequences analysed worldwide. Residues Asn or Asp (coded by aac or gac respectively) and Gly (coded by ggc) at aminoacid position 38 are consistently distinctive of vk210 and vk247 sequences, respectively. Moreover, the analysis of nucleotide sequences of parasites from around the world indicated that domains GGNA flanking the 3′ terminal CRR of vk210 are coded by alleles ggaggaaac/tgca/g (−ca or −tg), while those of vk247 are coded by a different allele (−tt) (Figure 3). Nucleotide substitutions were used to identify recombination arrangements, e.g., North Korean (EU401928.1) and Indonesian (EU401927.1) present on the amino terminus the residue Gly −38 (vk247 type), followed by 19 vk247 type repeat units and one vk210 type unit (RAT-III) or four vk247 type repeat units and 16 vk210repeat units, respectively. Additionally, the GGNA domain flanking the CRR at the carboxyl terminus resembled vk210 nucleotide sequences coded by allele −tg. This domain is flanked by the ANKKAEDA sequence. A previously described strain from Gabon presents a more complex sequence structure (vk247-vk210-vk247-vk210), had Gly −38, then 19 vk210 repeats followed by one vk247 repeat and the domain GGNA coded by allele −ca.

## Discussion

Large human population movements have taken place in ancient and recent times. They have facilitated the spread of infectious diseases, including malaria, throughout the world [49-53]. Within the Americas, current human population movements between countries are very active and comprise migrants from all around the world (passing through South and Mesoamerican regions) into North America [54-56]. This widespread phenomenon could favour the introduction of new malaria strains into new regions with favourable conditions for parasite transmission [11].

Molecular biology, phylogenetic and bioinformatics approaches were integrated to analyse *P. vivax csp* diversity and the genetic relationships of parasites from the Pacific Ocean coast of Latin America with other isolates of worldwide origin. As a screening approach, the PCR-RFLP results suggested that *csp* vk210 and vk247 genotypes had a non-homogeneous geographic distribution among the study areas. In Southern Mexico, both genotypes were present as previously reported [28], while in Nicaragua and Peru only vk210 and vk247 parasites were detected, respectively. Parasite sampling in Nicaragua was carried out in five different villages, suggesting a low prevalence or non-existence of *csp* vk24*7* parasites, at least during the year-round sampling effort. In agreement with these observations, 17 *P. vivax* samples collected during 2004 and 2005 in the North, North-West and South of Guatemala [57] and 84 isolates collected between 2010 and 2011 in 20 municipalities of Honduras [58] were all of the vk210 genotype. Similarly, the predominance of a single vk247 genotype in infections reported in a Colombian region close to the Peruvian border [31] concurs with the occurrence of this genotype in Piura, Peru. While no variation was detected among vk247 sequences, genetic variation within *csp* vk210 genotypes was detected in Southern Mexico and Nicaragua.

The PCR-RFLP analysis provided enough resolution to allow an initial classification of vk210 parasites. Six different vk210 RFLP subtypes were identified; four in Mexico and three in Nicaragua. Subtype vk210*a* was observed in both countries and was the most frequent, the *e* and *f* from Nicaragua were less frequent, and could be local variations from subtype vk210*a*. The better resolution was given by the nucleotide sequence to vk210*a*^*I*^ and *a*^*II*^ (detected only in Mexico) and subtype *a*^*III*^ (detected only in Nicaragua). As Alu I restriction enzyme digest the whole CRR, it is suggested the presence of other genotypes likely at the CRR of vk210*a*[59]. No PCR-RFLP polymorphism was detected in the vk247 isolates.

The integration of the DNA polymorphism examination, MD analysis of the CRR and the phylogenetic trees of the 3′ terminal nucleotide sequences provided a better understanding into the *P. vivax csp* genetic relationships of Mexican, Nicaraguan and Peruvian parasites with those from other geographic origins. The genetic diversity of the CSP gene was mostly restricted to the CRR and the 3′ terminal variable region, and was due to nucleotide changes and variability in the repeat numbers as reported in other studies. This gene has been useful in molecular epidemiological studies, understanding transmission dynamics and evolutionary relationships [13,14,60-63]. However, being under selective pressure, phylogenetic studies using this marker must be cautiously interpreted [15]. Moreover, the repetitive nature of the CRR is not amenable to traditional phylogenetic analysis. To overcome this limitation, we implemented the MD analysis as suggested by Hughes [13]. Although this analysis is not time calibrated, parameters such as average *p*, *prop. 0*, *prop. >0.25* and *σ* (*p*) suggest temporal relations in the occurrence of *rat* duplication/deletion events. The MD of CRR is in accordance with a relatively recent *rat* duplication and deletion as a possibly consequence of slipped strand mispairing during DNA replication, as previously suggested for *Plasmodium* repeat regions [14]. However, differences of the MD parameters enabled the identification of distinctive level of concerted evolution and geographical segregation, strongly supported by hierarchical clustering: 1) based on higher values of average *p*, *σ* (*p*) and *prop. >0.25* as well as the carboxyl end, vk247 isolates form a unique cluster with no geographical correlation; 2) vk210 parasites could be subdivided into three distinct clusters (C1 (A and B) and C2) that partially correlated with the RFLP genotype, and where C1 cluster was composed mainly from LA isolates, whereas C2 was composed mainly by OA isolates; 3) phylogenetic similarities of the 3′ terminal region, particularly the allele coding for GGNA domain and the presence or absence of the ANKKAEDA in C2 and C1, respectively, supported MD analysis based sub-clustering; and 4) the difference of the MD parameters between vk247 and vk210 may reflect the different levels of concerted evolution. Considerations that address each of the four points listed above are as follows:

All vk247 sequences cluster into a single group by MD parameters and all of them express at least one copy of the ANKKAGDA domain at the carboxyl end. The vk247 sequences from different distant locations (Oaxaca and Chiapas) of Mexico and Peru are highly conserved and they are identical, both at CRR and the 3′ terminal end, to that of Iranian parasites [47] and highly similar to Colombian parasites (JN689931.1, JN689932.1, [31]). It is noteworthy that a divergence occurs between *rats* of the CRR of Mexican, Colombian and Iranian parasites compared to those of Brazilian and Thai isolates (Figure 4). While Brazilian and Thai isolates have the same genotype at the 3′ terminal end, they seem to have undergone different differentiation processes at the CRR. Zakeri *et al.*[47] suggested that an extra copy of the domain GGQAAGGNAANKKAGDAGA at the variable carboxyl end was originated by a nucleotide fragment duplication by replication slippage, which includes five nucleotides of the last repeat up to the A residue that flanks at the GQGQ conserved domain. Such duplication is unique and increased the inter CRR-3′ terminal region variability. Further investigation would be necessary to determine the origin and dispersal of this genotype. The large *csp* polymorphism in vk247 parasites from the Pacific Ocean coast of Colombia [31] and Iran [47] is probably associated with prolonged and/or more complex transmission patterns of this genotype in these regions. The *csp* genotype vk247, first reported in Mexico and Peru in 1992 [24] might have been restricted to some locations and probably spread through the foothills of Southern Mexico after the environmental disturbances caused by hurricane Paulina in 1997 [19,28]. This hypothesis is consistent with microsatellite analysis of parasites collected in this region between 1997–2005, which revealed three populations with significant genetic differentiation and suggested that at least one population (f1 and/or f2) was recently introduced into the foothills [64]. The f1 seem to comprise parasites vk210 detected from 2001 and onwards and f2 comprised the vk247 [19,64].

Although MD analysis does not directly provide information on the type and position of nucleotide changes, it is outstanding that the main vk210 hierarchical clustering was strongly supported by the 3′ terminal polymorphism analysis. Two LA clusters of similar *p* distinguished by their (*σ (p)*) (C1A and C1B respectively) and one mainly OA of higher *p* (C2). Accordingly, higher numbers of exclusive repeat units were detected in OA than in LA isolates. In fact, LA isolates of cluster C1A (Mxch: 1–3, Nichn, Nir1, Sal I and Honduras III and South American isolates) display very limited *rat* variation (lower *σ* (*p*), *prop. >0.25* and *skew* values) and probable a more recent repeat duplication than C1B that includes Nichg and Mxch13 and other South American isolates, that probably result from an independent introduction to the Americas and a consequent population bottleneck. These results further expand those from a hypo-endemic region of Brazil [14] as result of clonal mode of reproduction especially when a low rate of mixed genotype infection prevails [14,15]. In Southern Mexico, the systematic blood collection during 2006–2008 revealed more than 90% single *csp* genotype infections similar to all parasite samples from Nicaraguan and Peru.

The similarity of the 3′ terminal nucleotide sequence among *csp* vk210 *a*^*I*^ and *a*^*II*^ of Mexican and all Nicaraguan isolates of C1 are consistent with previously reported LA isolates [14,31,41]. In contrast, the MD parameters of the CRR from Mexican sequences vk210*d*, *c* and *d* grouped them closely to OA isolates of higher variation (*p* and *σ*) than those from LA. Additionally, these sequences formed a closely related group, alleles vk210*b* and *d*, had almost identical CRR, and vk210*c* allele could likely be the result of a single or various deletion events from vk210*b*. In agreement with the CRR, the domain ANKKAEDA at the carboxyl end of these isolates indicated a close relationship to Asian parasites. In fact, the phylogenetic tree confirmed that the 3′ terminal region of the *csp* of these parasites was highly similar to those from various OA locations, including China, North Korea (NK), Philippines [65], Thailand [66], South Korea [67], and Iran [47]. Mitochondrial genome sequencing analysis suggests that these parasites are likely the result of an ancient population expansion in those regions [8].

Successful transmission of the parasite depends basically on the competence of the mosquito vectors present in each geographic region [6,19,68]. The recent predominance of both *csp* genotypes (vk247 and vk210) at similar frequencies in the foothills of Southern Mexico could be explained by the susceptibility of *Anopheles pseudopuntipennis,* the main vector in this region [19,28,64]. This vector is also present in Piura, Peru. While *An. Albimanus* is likely the main vector on the coastal regions of Mexico and Central America [19,28,58,69]. Interestingly, two *P. vivax* lineages (Old and New World) were previously proposed, based on mosquito susceptibility and ribosomal gene isoforms [6]. Extensive work only on the ribosomal variation with Colombian and Indian isolates partially contradicted the existence of such dichotomy [7]. It is possible to speculate that at least the isolates classified by Li et al. [6] as “New World” might be related to the C1A cluster, a subgroup of the 18S ribosomal RNA type Sal-I likely adapted to local *An. albimanus*. But further studies on vector susceptibility and parasite genomics are necessary to clarify of these conflicting results.

According to Hughes [13] the higher mismatch distribution values (*average p*, *σ* (*p*) and *prop. >0.25*) of all vk247 repeats (including the three divergent CRR types) suggest that they are likely more ancestral than vk210. Furthermore, the vk247 genotype displays high identity at the carboxyl end to the *csp* sequence reported for *Plasmodium cynomolgi* (Gen Bank AB524342.1). This contrasts with the higher *rat* diversity of vk210 than vk247 CRRs, possibly related to the higher worldwide prevalence of vk210 than vk247 [14,15,20,30,47,58,70-74]. In Southern Mexico vk247 became more prevalent from 1997 onwards [19,28]. However, the possibility that the vk247 *rats* are genetically more stable (i.e., less prone to replication slippage) than vk210 *rats* cannot be excluded.

The genome sequence analysis of *P. vivax* strains, including Sal I, Peru, Brazil I, India VII, North Korea and Mauritania [11,46], partially agrees with the MD of CRR analysis and the separation of clusters LA and OA isolates, and supported by the presence of higher number of private repeat units in OA isolates than in LA. However, more extensive genome sequencing analysis is required for a better insight into parasite evolution and its dispersal into Meso and South America. The marked genetic structure determined in LA regions, but the microheterogeneity [11,64,67,74,75] revealed the presence of scenarios epidemiologically diverse. *P. vivax* genetics and biological characteristics, host immunity and local vectors may contribute to their different patterns of demographic expansion favoured or limited by the eco-epidemiological conditions.

In Southern Mexico the low frequent genotypes vk210*b*, *c* and *d*, first time reported in America, and the lack of vk247 polymorphism suggest, a relatively recent introduction and/or expansion events of these genotypes at least into this region. Between 1970 and 1985, a constant decline in malaria cases, transmission focalization, and likely a limited gene flow between microregions occurred as a result of intensified control measures [76]. Genotype vk210*d* was detected by an intensify sampling between 2006 and 2008, and although vk210*b* and *c* were more prevalent between 2002 and 2005, their prevalence declined in 2006–2008, possible due to a significant reduction of malaria incidence in 2005.

This study contributes to the molecular epidemiology of *P. vivax* in the Americas, suggesting that the introduction of parasite’s genotypes vk247, vk210*b*, *c* and *d* (OA like) and LA clusters C1A and/or C1B might have occurred at different time points [77,78]. Whether they were introduced by ancestral migration events or more recent ones (pre- or during colonization of the American continent) [8,11,77,78] is beyond the scope of the present work. A recent study suggest that some Central American *P. vivax* were the most recently introduced, with TMRCA of 23 Kya, as expected were related to Mid-Eastern, Asian and South American parasites, their TMRCA of 158, ≥199 and 309 Kya were calculated, respectively, and several events of *P. vivax* introductions into South America were suggested [78]. Similarly, Yalcindag *et al.*[79] reported that *P. falciparum* were introduced into South America by various independent events from Africa by the slave trade. It emphasizes the need to extend and intensify genetic studies in the affected regions; a larger sample in more LA locations is required for a better understanding of their genetic epidemiology and evolutionary relationships, their transmission patterns and dispersion. The availability of the genome sequences of many *P. vivax* isolates [10,11,46] and the analysis of the haplotype structure will help to reveal the complex distribution of this parasite in Latin America, as well as the relationships with the worldwide genetic diversity.

## Abbreviations

CSP: Circumsporozoite protein; csp: Circumsporozoite gene; CRR: Central repeat region; MD: Mismatch distribution; RAT: Repeat allelic amino acid type; rat: Repeat allelic nucleotide type; domains RI and RII: Conserved regions I and II at the 5′ and 3′ terminal of the csp gene, respectively; LA: Latin America; OA: Outside Latin America.

## Competing interests

The authors declare that they have no competing interests.

## Authors’ contributions

LGC conceived, designed, deployed the study, and participated in data analysis, preparation of figures and tables and interpretation, and drafted the manuscript. JMB carried out the mismatch distribution analysis and hierarchical clustering, organized information in figures and tables, interpreted data and drafted the manuscript. CMS and FS participated in RFLP analysis and PCR amplification and cloning, nucleotide sequence alignment and phylogenetic analysis, and drafted the manuscript. AS and OCH participated in study design and sample collection of *P. vivax* samples from Chinandega, Nicaragua, its storage and drafted the manuscript. MHR participated in data organization and interpretation and drafted the manuscript. BE participated in study design and collection of *P. vivax* samples from Peru, and drafted the manuscript. All authors read and approved the final manuscript.

## Supplementary Material

Additional file 1 **A. Mismatch distribution analysis of the *P. vivax *circumsporozoite: CRR-vk210 from different geographic origins.** Click here for file

Additional file 2 **B. Mismatch distribution analysis of the *P. vivax *circumsporozoite CRR-vk247 from different geographic origins.** Click here for file

Additional file 3 **A. Frequency of amino acid and nucleotide repeat types in the circumsporozoite CR sequence, of the *P. vivax *vk210 from Latin America and outside America.** Click here for file

Additional file 4 **B. Frequency of amino acid and nucleotide repeat types in the circumsporozoite CR sequence, of the *P. vivax *vk247 from Latin America and outside America.** Click here for file
