# Supplementary material for: Molecular epidemiology of Plasmodium vivax in Latin America: polymorphism and evolutionary relationships of the circumsporozoite gene
Source: Malar J. 2013 Jul 15;12:243. doi: 10.1186/1475-2875-12-243 (PMC3729580; doi:10.1186/1475-2875-12-243)
Supplement: Additional file 1 — A. Mismatch distribution analysis of the P. vivax circumsporozoite: CRR-vk210 from different geographic origins. [file 1475-2875-12-243-S1.docx]

Additional file 1A. Mismatch distribution analysis of the *P. vivax* circumsporozoite

CRR-vk210 from different geographic origins.

| Country  of origin | | Strain/isolate | #RU | *Average p* | *Prop= 0* | *Prop > 0.25* | Skewness | GenBank TM |  |
| --- | --- | --- | --- | --- | --- | --- | --- | --- | --- |
| Brazil | O13B-2 | | 18 | 0.054 | 0.203 | 0 | 0.276 | DQ978675.1 | |
| Colombia | Nr03 | | 20 | 0.058 | 0.307 | 0 | 1.462 | GU339072.1 | |
| Mexico | Mxch3-vk210a^I^ | | 20 | 0.068 | 0.17 | 0 | -0.207 | JQ511265.1 | |
| Honduras | Honduras III | | 20 | 0.068 | 0.17 | 0 | -0.207 | DQ156131.1 | |
| Colombia | Pt04.1 | | 20 | 0.069 | 0.144 | 0 | -0.202 | GU339085.1 | |
| Gabon | G24 | | 19 | 0.069 | 0.129 | 0 | 0.087 | ^a^U09737.1 | |
| Brazil | Brazil IV | | 18 | 0.069 | 0.19 | 0.026 | 1.384 | FJ845386.1 | |
| Nicaragua | Nir1-vk210*a^III^* | | 22 | 0.07 | 0.209 | 0 | 0.016 | JQ511282.1 | |
| El Salvador | Sal I | | 20 | 0.071 | 0.15 | 0 | -0.256 | XM_001613018 | |
| Brazil | O10 | | 19 | 0.072 | 0.205 | 0.018 | 1.099 | DQ978656.1 | |
| Brazil | O4 | | 18 | 0.073 | 0.163 | 0.033 | 1.292 | DQ978651.1 | |
| Nicaragua | Nichg-vk210*f* | | 18 | 0.074 | 0.17 | 0.013 | 1.107 | JQ511284.1 | |
| Nicaragua | Nichn-vk210*e* | | 19 | 0.075 | 0.111 | 0 | -0.312 | JQ511285.1 | |
| Brazil | Brazil (VII) | | 19 | 0.075 | 0.193 | 0.018 | 1.001 | FJ845389.1 | |
| Brazil | Brazil I clone I | | 21 | 0.077 | 0.173 | 0 | -0.474 | DQ156132.1 | |
| Brazil | Brazil (VIII) | | 21 | 0.077 | 0.129 | 0 | -0.467 | FJ845391 .1 | |
| Brazil | Brazil I | | 19 | 0.077 | 0.105 | 0 | -0.41 | ^b^EU401924.1 | |
| Brazil | Belem | | 20 | 0.077 | 0.17 | 0 | 1.082 | M11926.1 | |
| Brazil | I7 | | 19 | 0.077 | 0.15 | 0 | 1.091 | DQ978657.1 | |
| Brazil | I3 | | 19 | 0.077 | 0.123 | 0.012 | 1.35 | DQ978658.1 | |
| Brazil | *P. simium* I | | 21 | 0.078 | 0.119 | 0.014 | 1.104 | L05068.1 | |
| Mexico | Mxch13-vK210*a^II^* | | 20 | 0.079 | 0.17 | 0 | 1.05 | JQ511280.1 | |
| Brazil | Brazil (VII) | | 20 | 0.079 | 0.137 | 0.011 | 1.05 | FJ845390.1 | |
| Mexico | Mxch14-vk210*d* | | 18 | 0.079 | 0.143 | 0.013 | 1.03 | US | |
| Iran | P28 | | 18 | 0.08 | 0.131 | 0.013 | 1.13 | AY632256.1 | |
| India | India (03-1) | | 18 | 0.08 | 0.131 | 0.013 | 1.13 | DQ156140.1 | |
| India | NE12 | | 19 | 0.081 | 0.118 | 0 | 1.591 | FJ491119.1 | |
| Philippines | PH-46 | | 18 | 0.081 | 0.124 | 0.033 | 1.281 | U08980.1 | |
| Vietnam | Palo alto c1 (Vietnam IV) | | 16 | 0.081 | 0.103 | 0.066 | 1.493 | ^c, d^EU401929.1 | |
| Mexico | Mxch4-VK210*b* | | 18 | 0.082 | 0.15 | 0.013 | 0.966 | JQ511267.1 | |
| Iran | P59 | | 18 | 0.082 | 0.144 | 0.013 | 1.041 | AY632287.1 | |
| Solomon | SOL-83 | | 18 | 0.082 | 0.118 | 0.013 | 1.13 | ^e^U08982.1 | |
| Iran | CHPVCS7 | | 18 | 0.082 | 0.098 | 0.013 | 1.152 | AY443706.1 | |
| Indonesia | Indonesia XIX | | 16 | 0.082 | 0.117 | 0.017 | 1.16 | ^d^EU401927.1 | |
| Iran | CHPVCS21 | | 18 | 0.084 | 0.111 | 0.013 | 1.097 | AY443720.1 | |
| Mauritania | Mauritania | | 18 | 0.084 | 0.111 | 0.013 | 1.097 | AY674050.1 | |
| Iran | P9 | | 18 | 0.084 | 0.105 | 0.013 | 1.112 | AY367286.1 | |
| North Korea | KNIH | | 18 | 0.084 | 0.111 | 0.02 | 0.937 | AF316581.1 | |
| South Korea | KPVCSP01-22 | | 18 | 0.084 | 0.111 | 0.02 | 0.937 | DQ859754.1 | |
| India | NE10 | | 18 | 0.084 | 0.118 | 0.039 | 1.195 | FJ491117.1 | |
| Thailand | PFAC1RPTB | | 18 | 0.084 | 0.137 | 0.085 | 1.641 | M28746.1 | |
| India | India VII | | 16 | 0.086 | 0.117 | 0.025 | 1.084 | ^d^EU401926.1 | |
| Iran | KPVCS6 | | 17 | 0.087 | 0.096 | 0.015 | 1.084 | AY632325.1 | |
| Iran | P1 | | 16 | 0.088 | 0.092 | 0.017 | 1.033 | AY367278.1 | |
| Korea | Korea (02-9) | | 18 | 0.088 | 0.085 | 0.02 | 0.89 | DQ156137.1 | |
| Indonesia | Indonesia VII | | 18 | 0.088 | 0.111 | 0.02 | 0.991 | DQ156135.1 | |
| Mexico | Mxch3-vK210*c* | | 12 | 0.092 | 0.152 | 0.015 | 0.755 | JQ511266.1 | |
| Iran | KPVCS1 | | 17 | 0.092 | 0.074 | 0.015 | 1.027 | AY632320.1 | |
| New Guinea | Chesson I | | 16 | 0.094 | 0.183 | 0.017 | 0.398 | ^d^EU401925.1 | |
| India | ND3 | | 13 | 0.094 | 0.192 | 0.038 | 0.564 | FJ491100.1 | |
| North Korea | KNIH | | 12 | 0.102 | 0.061 | 0.03 | 0.717 | AF316580.1 | |
| India | NEO10 | | 10 | 0.102 | 0.044 | 0.089 | 0.935 | FJ491128.1 | |
| South Korea | KPVCSP02-20 | | 20 | 0.105 | 0.092 | 0 | 0.478 | DQ859760.1 | |
| North Korea | PFACSCA | | 20 | 0.111 | 0.137 | 0 | 0.194 | M20670.1 | |
| South Korea | KPVCSP02-39 | | 20 | 0.135 | 0.098 | 0 | -0.273 | DQ859768.1 | |
|  |  | |  |  |  |  |  |  | |

Data were listed by *P* and *prop. > 0.25.* Those from Mexico and Nicaragua are underlined.

Not included in the analysis:

^a^ the repeat unit ANGAGGQAA (vk247) flanking the carboxyl region.

Amino acid segments ^b^(GNGRAAGQAAGGNRAAGQADA) and ^c^(GGNGAGGQAEP) flanking the 3´terminal region.

^d^ four vk247 repeat units flanking the RI domain..

^e^ an incomplete repeat VDRADGQP flanking Region I.

P = average proportion of nucleotide difference between pairs of repeat units in the same *csp* tandem repeat

*Prop. 0* = proportion of pairwise comparisons of nucleotide sequences for which *p* = 0

*Prop.*>*25* = proportion of pairwise comparisons of nucleotide sequences for which *p* > 0.25

Skewness of the distribution of *p* values for all pairwise comparisons of nucleotide sequences

#RU, number of central repeat units.
