# Supplementary material for: Molecular epidemiology of Plasmodium vivax in Latin America: polymorphism and evolutionary relationships of the circumsporozoite gene
Source: Malar J. 2013 Jul 15;12:243. doi: 10.1186/1475-2875-12-243 (PMC3729580; doi:10.1186/1475-2875-12-243)
Supplement: Additional file 2 — B. Mismatch distribution analysis of the P. vivax circumsporozoite CRR-vk247 from different geographic origins. [file 1475-2875-12-243-S2.docx]

Additional file 1B. Mismatch distribution analysis of the *P. vivax* circumsporozoite CRR-vk247 from different geographic origins.

| Country  of origin | Strain/isolate | #RU | | *averagep* | | *Prop= 0* | *prop > 0.25* | Skewness | GenBank TM | | |
| --- | --- | --- | --- | --- | --- | --- | --- | --- | --- | --- | --- |
| Thailand | PFAC1RPTA | | 20 | | 0.078 | 0.175 | 0.095 | 2.234 | M28745.1 | | |
| Colombia | Pt03 | | 18 | | 0.088 | 0.137 | 0.092 | 1.44 | GU339084.1 | | |
| Colombia | Vc05 | | 18 | | 0.089 | 0.131 | 0.092 | 1.41 | GU339079.1 | |  |
| Colombia | Ch03 | | 20 | | 0.091 | 0.163 | 0.084 | 1.224 | GU339067.1 | |  |
| Bangladesh | Bangladesh | | 26 | | 0.092 | 0.185 | 0.065 | 1.157 | AY843440.1 | |  |
| Brazil | B7-4 | | 20 | | 0.092 | 0.153 | 0.068 | 1.117 | M69062.1 | |  |
| Colombia | Ch04 | | 18 | | 0.092 | 0.157 | 0.092 | 1.168 | GU339068.1 | |  |
| Vietnam | VietnamVI | | 21 | | 0.093 | 0.138 | 0.081 | 1.396 | DQ156141.1 | |  |
| Colombia | Ch01 | | 18 | | 0.093 | 0.137 | 0.092 | 1.221 | GU339065.1 | |  |
| Colombia | Vc01 | | 20 | | 0.094 | 0.153 | 0.084 | 1.163 | GU339075.1 | |  |
| Iran | CHPVCS11 | | 20 | | 0.095 | 0.147 | 0.079 | 1.329 | AY443710.2 |  |  |
| Brazil | *P. simium* II | | 21 | | 0.095 | 0.157 | 0.071 | 1.118 | L05069.1 |  |  |
| Colombia | Ca05 | | 20 | | 0.095 | 0.132 | 0.084 | 1.186 | GU339064.1 |  |  |
| Iran | CHPVCS33 | | 20 | | 0.096 | 0.116 | 0.079 | 1.251 | AY632294.2 |  |  |
| Colombia | Pt02.1 | | 18 | | 0.096 | 0.131 | 0.092 | 1.291 | GU339082.1 |  |  |
| Iran | CHPVCS37 | | 20 | | 0.097 | 0.158 | 0.079 | 1.089 | AY632298.2 |  |  |
| Iran | CHPVCS55 | | 19 | | 0.098 | 0.158 | 0.082 | 1.11 | AY632316.1 |  |  |
| Mexico/Peru | Mxch6/PeruI | | 19 | | 0.098 | 0.146 | 0.088 | 1.105 | JQ511270.1/ JQ511286.1 |  |  |
| Colombia | Vc02 | | 20 | | 0.099 | 0.132 | 0.074 | 1.041 | GU339076.1 |  |  |
| Colombia | Ca04 | | 20 | | 0.1 | 0.132 | 0.074 | 1.042 | GU339063.1 |  |  |
| Vietnam | Palo alto c2 | | 20 | | 0.1 | 0.09 | 0.08 | 1.38 | EU401930.1 |  |  |
| Colombia | Nr02 | | 20 | | 0.101 | 0.105 | 0.074 | 1.083 | GU339071.1 |  |  |
| Colombia | Vc04 | | 18 | | 0.101 | 0.092 | 0.085 | 1.155 | GU339078.1 |  |  |
| Colombia | Nr01 | | 20 | | 0.104 | 0.132 | 0.068 | 0.821 | GU339070.1 |  |  |
| North Korea | North Korea | | 19 | | 0.104 | 0.117 | 0.105 | 1.22 | ^a^EU401928.1 |  |  |
| Iran | CHPVCS38 | | 20 | | 0.106 | 0.095 | 0.079 | 1.096 | AY632299.2 |  |  |
| Colombia | Ch05 | | 20 | | 0.106 | 0.111 | 0.079 | 1.291 | GU339069.1 |  |  |

Data were listed by *P* and *prop. > 0.25.* That from Mexico and Peru are underlined, in addition isolates from Colombia (JN689931.1) and Iran (AY632330.1) showed the same nucleotide sequence. ^a^ the repeat unit GNGAGGQAA (vk210), flanking the 3´terminal region was not included in the analysis.

*P* = average proportion of nucleotide difference between pairs of repeat units in the same *csp* tandem repeat

*Prop. 0* = proportion of pairwise comparisons of nucleotide sequences for which *p* = 0

*Prop.*>*25* = proportion of pairwise comparisons of nucleotide sequences for which *p* > 0.25

Skewness of the distribution of *p* values for all pairwise comparisons of nucleotide sequences

#RU, number of central repeat units.
