# Supplementary material for: Molecular epidemiology of Plasmodium vivax in Latin America: polymorphism and evolutionary relationships of the circumsporozoite gene
Source: Malar J. 2013 Jul 15;12:243. doi: 10.1186/1475-2875-12-243 (PMC3729580; doi:10.1186/1475-2875-12-243)
Supplement: Additional file 3 — A. Frequency of amino acid and nucleotide repeat types in the circumsporozoite CR sequence, of the P. vivax vk210 from Latin America and outside America. [file 1475-2875-12-243-S3.docx]

Additional file 2A. Frequency of amino acid and nucleotide repeat types in the circumsporozoite CR sequence, of the *P. vivax* vk210 from Latin America and outside America.

| **RAT No.** | **Amino acid sequence** | *rat´*s code | Nucleotide sequence | Number (n) *rats* per group: | | |  |
| --- | --- | --- | --- | --- | --- | --- | --- |
|  |  |  |  | Overall^a^  n | Latin America^b^  n (%) | Outside America^c^  n (%) | Chi square  (CI95%) |
| **I** | **GDRADGQPA** | I.1 | GGAGACAGAGCAGATGGACAGCCAGCA | 168 | 123 ^d^ | 45 | *p=0.0001* |
|  |  | I.2 | GGCGATAGAGCAGATGGACAGCCAGCA | 90 | 32 | 58 ^d^ | *p=0.0001* |
|  |  | I.4 | GGTGATAGAGCAGATGGACAGCCAGCA | 44 | 13 | 31 ^d^ | *p=0.0001* |
|  |  | I.3 | GGAGATAGAGCAGATGGACAGCCAGCA | 52 | 45 ^d^ | 7 | *p=0.0001* |
|  |  | I.6 | GGAGATAGAGCAGATGGACAACCAGCA | 34 | 15 | 19 |  |
|  |  | I.5 | GGTGATAGAGCAGATGGACAACCAGCA | 37 | 30 ^d^ | 7 | *P=0.008* |
|  |  | I.7 | GGAGACAGAGCAGATGGACAACCAGCA | 8 | 6 | 2 |  |
|  |  | I.8 | GGAGACAGAGCAGATGGTCAGCCAGCA | 2 | 1 | 1 |  |
|  |  | I.9 | GGCGATAGAGCAGATGGACAACCAGCA | 1 | 0 | 1 |  |
|  |  | I.10 | GGCGATAGAGCAGATGGCCAGCCAGCG | 1 | 0 | 1 |  |
|  |  | I.11 | GGTGATAGAGCAGATGGACAGCCAGCG | 1 | 0 | 1 |  |
|  |  |  | **subtotal I:** | **438** | **265** ^e^ | **173** | ***p=0.0001*** |
| **II** | **GDRAAGQPA** | II.1 | GGAGATAGAGCAGCTGGACAGCCAGCA | 222 | 84 | 138 |  |
|  |  | II.2 | GGTGATAGAGCAGCTGGACAACCAGCA | 65 | 19 | 46 |  |
|  |  | II.3 | GGAGATAGAGCAGCTGGACAACCAGCA | 39 | 15 | 24 |  |
|  |  | II.5 | GGCGATAGAGCAGCTGGACAGCCAGCA | 21 | 1 | 20 ^d^ | *p=0.002* |
|  |  | II.4 | GGTGATAGAGCAGCTGGACAGCCAGCA | 25 | 15 | 10 |  |
|  |  | II.6 | GGCGATAGAGCAGCTGGACAACCAGCA | 14 | 0 | 14 ^d^ | *p=0.003* |
|  |  | II.7 | GGAGATAGAGCAGCTGGACAGCCAGCG | 1 | 0 | 1 |  |
|  |  | II.8 | GGAGATAGAGCAGCTGGTCAGCCAGCA | 2 | 0 | 2 |  |
|  |  | II.9 | GGAGACAGAGCAGCTGGACAGCCAGCA | 2 | 2 | 0 |  |
|  |  | II.10 | GGAGATAGAGCAGCCGGACAGCCAGCA | 1 | 0 | 1 |  |
|  |  | II.11 | GGAGATAGAGCAGCTGGTCAACCAGCA | 1 | 0 | 1 |  |
|  |  | II.12 | GGTGATAGAGCAGCTGGTCAGCCAGCA | 1 | 0 | 1 |  |
|  |  | II.13 | GGAGATAGAGCAGCTGGACAGCCAGCT | 1 | 0 | 1 |  |
|  |  |  | **Subtotal II:** | **394** | **136** | **259** ^e^ | ***p=0.0001*** |
| **III** | **GNGAGGQAA** | III.1 | GGAAATGGTGCAGGTGGACAGGCAGCA | 45 | 13 | 32 |  |
|  |  | III.2 | GGAAATGGTGCAGGTGGACAGGCAGCG | 1 | 0 | 1 |  |
|  |  | III.3 | GGAAATGGTGCAGGTGGACAGGCCGCA | 1 | 0 | 1 |  |
|  |  |  | **Subtotal III:** | **47** | **13** | **34** ^e^ | ***p=0.010*** |
| **IV** | **GDRAAGQAA** | **IV** | GGAGATAGAGCAGCTGGACAGGCAGCA | **29** | **23** | **6** | ***p=0.0001*** |
| **V** | **GDGAAGQPA** | V.1 | GGCGATGGAGCAGCTGGACAGCCAGCA | 24 | 0 | 24 |  |
|  |  | V.2 | GGCGATGGAGCAGCTGGACAGCCAGCT | 1 | 0 | 1 |  |
|  |  |  | **Subtotal V:** | **25** | **0** | **25** ^e^ | ***p=0.0001*** |
| **VI** | **GNGAGGQPA** | VI.1 | GGAAATGGTGCAGGTGGACAACCAGCA | 3 | 0 | 3 |  |
|  |  | VI.2 | GGAAATGGTGCAGGTGGACAGCCAGCA | 3 | 0 | 3 |  |
|  |  |  | **Subtotal VI:** | **6** | **0** | **6** ^e^ | ***p=0.023*** |
| **VII** | **GDRADGQAA** | VII.1 | GGAGACAGAGCAGATGGACAGGCAGCA | 2 | 0 | 2 |  |
|  |  | VII.2 | GGCGATAGAGCAGATGGACAGGCAGCA | 1 | 0 | 1 |  |
|  |  |  | **Subtotal VII:** | **3** | **0** | **3** | **NS** |
| **VIII** | **GDRAPGQPA** |  | GGCGATAGAGCACCTGGACAGCCAGCA | **2** | **0** | **2** | **NS** |
| **IX** | **GDRADGHPA** |  | GGAGATAGAGCAGATGGACACCCAGCA | **1** | **0** | **1** | **NS** |
| **X** | **GDRADEQPA** |  | GGCGATAGAGCAGATGAACAGCCAGCA | **1** | **0** | **1** | **NS** |
| **XI** | **GDSADGQPA** |  | GGCGATAGTGCAGATGGACAGCCAGCA | **1** | **0** | **1** | **NS** |
| **XII** | **GDRAGGQAA** |  | GGAGATAGAGCAGGTGGACAGGCAGCA | **1** | **1** | **0** | **NS** |
| **XIII** | **GNRAAGQAA** |  | GGAAATAGAGCAGCTGGACAGGCAGCA | **1** | **1** | **0** | **NS** |
| **XIV** | **GNRADGQPA** |  | GGAAATAGAGCAGATGGACAGCCAGCA | **1** | **1** | **0** | **NS** |
| **XV** | **GDGAAGQPA** |  | GGTGATGGAGCAGCTGGACAACCAGCA | **1** | **0** | **1** | **NS** |
| **XVI** | **DDRAAGQPA** |  | GACGATAGAGCAGCTGGACAACCAGCA | **1** | **0** | **1** | **NS** |
| **XVII** | **GDRAAGRPA** |  | GGAGATAGAGCAGCTGGACGGCCAGCA | **1** | **0** | **1** | **NS** |
| **XVIII** | **GDGADGQPA** |  | GGTGATGGAGCAGATGGACAGCCAGCA | **1** | **0** | **1** | **NS** |
| **XIX** | **GDGAAVQPA** |  | GGTGATGGAGCAGCTGTACAACCAGCA | **1** | **0** | **1** | **NS** |
| **XX** | **GDRPAGQPA** |  | GGCGATAGACCAGCTGGACAGCCAGCA | **1** | **0** | **1** | **NS** |
|  |  |  | **TOTAL:** | **957** | **440** | **517** |  |

a, All 53 isolates worldwide

b, 30 isolates

c, 23 isolates

d, shows a *rat* (nucleotide) number that codes for one high prevalent RAT (amino acid) significantly higher for one geographic region.

e, shows a RAT(amino acid) number significantly higher for one geographic region; data are shown in bold letters.

NS, no statistical significance
