# Supplementary material for: Molecular epidemiology of Plasmodium vivax in Latin America: polymorphism and evolutionary relationships of the circumsporozoite gene
Source: Malar J. 2013 Jul 15;12:243. doi: 10.1186/1475-2875-12-243 (PMC3729580; doi:10.1186/1475-2875-12-243)
Supplement: Additional file 4 — B. Frequency of amino acid and nucleotide repeat types in the circumsporozoite CR sequence, of the P. vivax vk247 from Latin America and outside America. [file 1475-2875-12-243-S4.docx]

Additional file 2B. Frequency of amino acid and nucleotide repeat types in the circumsporozoite CR sequence, of the *P. vivax* vk247 from Latin America and outside America.

| **RAT No.** | **Amino acid sequence** | *rat´*s code | Nucleotide sequences | Number of *repeat units* per group (n) : | | | |
| --- | --- | --- | --- | --- | --- | --- | --- |
|  |  |  |  | Overall ^a^  n | Latin America ^b^  n | Outside America ^c^  n | Chi square  (CI95%) |
| **I** | **ANGAGNQPG** | I.1 | GCAAATGGGGCTGGCAATCAACCAGGA | 128 | 66 | 62 |  |
|  |  | I.2 | GCAAATGGGGCAGGCAATCAACCAGGA | 109 | 76 ^d^ | 33 | *P=0.005* |
|  |  | I.3 | GCAAATGGGGCAGGTAATCAACCAGGA | 79 | 53 | 26 |  |
|  |  | I.4 | GCAAATGGGGCCGGCAATCAACCAGGA | 29 | 13 | 16 |  |
|  |  | I.5 | GCAAATGGAGCAGGCAATCAACCAGGA | 7 | 0 | 7 ^d^ | *P=0.002* |
|  |  | I.6 | GCAAATGGAGCTGGAAATCAACCAGGA | 1 | 1 | 0 |  |
|  |  | I.7 | GCAAATGGGGCAGGCAATCAACCAGGT | 1 | 1 | 0 |  |
|  |  | I.8 | GCAAATGGGGCAGGTAATCAACCCGGA | 1 | 0 | 1 |  |
|  |  | I.9 | GCAAATGGGGCCGGTAATCAACCAGGA | 1 | 0 | 1 |  |
|  |  | I.10 | GCAAATGGGGCGGGCAATCAACCAGGA | 1 | 0 | 1 |  |
|  |  | I.11 | GCAAATGGGGCGGGTAATCAACCAGGA | 1 | 0 | 1 |  |
|  |  | I.12 | GCAAATGGGGCTGGCAATCAACCTGGA | 1 | 0 | 1 |  |
|  |  | I.13 | GCAAATGGGGCTGGCAATCAACCGGGA | 1 | 0 | 1 |  |
|  |  |  | **subtotal I:** | **360** | **210** | **150** | **NS** |
| **II** | **ANGAGDQPG** | II.1 | GCAAATGGAGCAGGTGATCAACCAGGA | 68 | 42 | 26 |  |
|  |  | II.2 | GCAAATGGGGCAGGTGATCAACCAGGA | 6 | 5 | 1 |  |
|  |  | II.3 | GCAAATGGAGCAGGCGATCAACCAGGA | 2 | 2 | 0 |  |
|  |  | II.4 | GCAAATGGGGCAGGCGATCAACCAGGA | 3 | 2 | 1 |  |
|  |  |  | **Subtotal II:** | **79** | **51** | **28** | **NS** |
| **III** | **ANGAGGQAA** | III.1 | GCAAATGGAGCAGGTGGACAGGCAGCA | 23 | 15 | 8 |  |
|  |  | III.2 | GCAAATGGAGCAGGTGGACAGGCGGCA | 1 | 1 | 0 |  |
|  |  | III.3 | GCAAATGGGGCAGGTGGACAGGCAGCA | 1 | 0 | 1 |  |
|  |  | III.4 | GCAAATGGTGCAGGTGGACAGGCAGCA | 1 | 0 | 1 |  |
|  |  |  | **Subtotal III:** | **26** | **16** | **10** | **NS** |
| **IV** | **EDGAGDQPG** | IV.1 | GAAGATGGGGCAGGTGATCAACCAGGA | 16 | 15 | 1 |  |
|  |  | IV.2 | GAAGATGGAGCAGGTGATCAACCAGGA | 2 | 0 | 2 |  |
|  |  | IV.3 | GAAGATGGAGCTGGTGATCAACCAGGA | 1 | 0 | 1 |  |
|  |  |  | **Subtotal IV:** | **19** | **16** | **4** | **NS** |
| **V** | **ANGADDQPG** | V.1 | GCAAATGGAGCAGATGATCAACCAGGA | 18 | 12 | 6 |  |
|  |  | V.2 | GCAAATGGAGCAGACGATCAACCAGGA | 1 | 1 | 0 |  |
|  |  |  | **Subtotal V:** | **19** | **13** | **6** | **NS** |
| **VI** | **EDGAGNQPG** |  | GAAGATGGGGCAGGCAATCAACCAGGA | **6** | **0** | **6** | **NS** |
| **VII** | **ANGAGGQPG** |  | GCAAATGGAGCAGGTGGACAACCAGGA | **1** | **1** | **0** | **NS** |
| **VIII** | **ANGAGKQPG** |  | GCAAATGGGGCAGGTAAACAGCCAGGA | **1** | **1** | **0** | **NS** |
| **IX** | **ANGARNQPG** |  | GCAAATGGGGCACGTAATCAACCAGGA | **1** | **0** | **1** | **NS** |
|  |  |  | **Total** | **512** | **307** | **205** |  |

a, All 26 isolates worldwide

b, 16 isolates

c, 10 isolates

d, shows a *rat* (nucleotide) number significantly higher, that codes for one high prevalent RAT (amino acid) in one geographic group.
